# Vitamin A bio-modulates apoptosis via the mitochondrial pathway after hypoxic-ischemic brain damage

**DOI:** 10.1186/s13041-018-0360-0

**Published:** 2018-03-13

**Authors:** Wei Jiang, Min Guo, Min Gong, Li Chen, Yang Bi, Yun Zhang, Yuan Shi, Ping Qu, Youxue Liu, Jie Chen, Tingyu Li

**Affiliations:** 1 Children Nutrition Research Center, Children’s Hospital of Chongqing Medical University, Chongqing, 400014 China; 2 Ministry of Education Key Laboratory of Child Development and Disorders, Chongqing, 400014 China; 3 China International Science and Technology Cooperation Base of Child Development and Critical Disorders, Chongqing, 400014 China; 4 Chongqing Key Laboratory of Translational Medical Research in Cognitive Development and Learning and Memory Disorders, Chongqing, 400014 China; 5 Children Rehabilitation Center, Children’s Hospital of Chongqing Medical University, Chongqing, China

**Keywords:** Vitamin A (VA), Hypoxic-ischemic brain damage (HIBD), Retinoic acid (RA), Apoptosis, Mitochondrial membrane potential (MMP), PI3K/Akt

## Abstract

Our previous studies demonstrated that vitamin A deficiency (VAD) can impair the postnatal cognitive function of rats by damaging the hippocampus. The present study examined the effects of retinoic acid (RA) on apoptosis induced by hypoxic-ischemic damage in vivo and in vitro*,* and investigated the possible signaling pathway involved in the neuroprotective anti-apoptotic effects of RA. Flow cytometry, immunofluorescence staining and behavioral tests were used to evaluate the neuroprotective and anti-apoptotic effects of RA. The protein and mRNA levels of RARα, PI3K, Akt, Bad, caspase-3, caspase-8, Bcl-2, Bax, and Bid were measured with western blotting and real-time PCR, respectively. We found impairments in learning and spatial memory in VAD group compared with vitamin A normal (VAN) and vitamin A supplemented (VAS) group. Additionally, we showed that hippocampal apoptosis was weaker in the VAN group than that in VAD group. Relative to the VAD group, the VAN group also had increased mRNA and protein levels of RARα and PI3K, and upregulated phosphorylated Akt/Bad levels in vivo. In vitro, excessively low or high RA signaling promoted apoptosis. Furthermore, the effects on apoptosis involved the mitochondrial membrane potential (MMP). These data support the idea that sustained VAD following hypoxic-ischemic brain damage (HIBD) inhibits RARα, which downregulates the PI3K/Akt/Bad and Bcl-2/Bax pathways and upregulates the caspase-8/Bid pathway to influence the MMP, ultimately producing deficits in learning and spatial memory in adolescence. This suggests that clinical interventions for HIBD should include suitable doses of VA.

**Electronic supplementary material:**

The online version of this article (10.1186/s13041-018-0360-0) contains supplementary material, which is available to authorized users.

## Introduction

Hypoxic-ischemic brain damage (HIBD) is the most common central nervous system diseases in the neonatal period and has a poor prognosis. A large number of children have residual nerve damage, which is manifested as mental developmental delay, intellectual disability, or even death [1–3]. Meanwhile, pediatric vitamin A deficiency (VAD) is a global public health problem. A preclinical investigation found that neonates with HIBD suffered from more severe VAD than those who had pneumonia or those who were healthy, and the vitamin A (VA) levels did not significantly increase with advancing age (Additional file 1: Figure S1). It has previously been found that VA could affect neural development after birth [4]. Therefore, it was hypothesized that VA can affect neural tissue and functional outcome after HIBD.

The hippocampus is crucial for learning and memory [5] and is susceptible to HIBD injury [6]. VA is an important fat-soluble vitamin that carries out physiological functions similar to those of hormones via its main derivative, retinoic acid (RA). The hippocampus and its surrounding meninges synthesize and metabolize RA, promoting the expression of retinol-binding protein (RBP) [7]. Apoptosis is the important mechanism of pathological damage in the acute stage of HIBD. Therefore, apoptosis in the hippocampus was the target of the present study.

VA plays a pivotal role in a suite of essential biologic processes as a powerful regulator of vision, reproduction, immunity, apoptosis, growth and development. RA is associated with cell proliferation and differentiation, and additionally contributes to the proper development of the vertebrate central nervous system [8, 9]. RA can modulate the transcription or nontranscription of downstream target genes or functional proteins through retinoic acid receptor (RAR)-mediated signal transduction. RAR heterodimers attach to specific DNA sequences or RA response elements (RAREs), which are typically composed of two direct repeats of a core hexameric motif. RA interacts with two major families of nuclear receptors: retinoic acid receptors (RAR) and retinoid X receptors (RXR). Each family is composed of three isotypes: α, β, and γ. The RARα isoform has an essential role in brain development and modulates adult brain function [10, 11].

RA can promote carcinoma cell apoptosis, and larger doses of all-trans retinoic acid are currently used for the therapy of certain cancers [12]. VAD causes apoptosis of pancreatic beta-cell masses [13]. Paradoxically, RA has been reported to have protective effects against the neuronal apoptosis caused by injury, and it enhances proliferation and survival. These effects all depend on transcriptional signaling that involves RA and anti-apoptosis pathways [14]. A previous study found that RARα was primarily a nuclear receptor present in the rat cerebral cortex and white matter during postnatal development [4]. VAD in pregnancy can attenuate the expression of RARα, causing concomitant deficits in active learning and spatial memory function in adolescence [4, 15]. It has been demonstrated that treatment with appropriate concentrations of RA can influence the mitochondrial membrane potential (MMP) to reduce the apoptosis of oxygen-glucose deprivation (OGD)-injured PC12 cells, possibly through the regulation of RARα signaling [16, 17]. It is speculated that the bidirectional regulation of apoptosis depends on the concentration of RA and the types of target cells and tissues. Accordingly, it is hypothesized that a suitable concentration of RA will have an anti-apoptotic effect on neurons in HIBD. Numerous studies have been devoted to investigating the mechanism of apoptosis and the pathway to antagonize hippocampal cell apoptosis after hypoxic-ischemic injury. However, these studies on hypoxic-ischemic damage and the potential mechanism of anti-apoptotic effects involved in RA are still inconclusive.

The present study examined the effects of RA on apoptosis produced by hypoxic-ischemic damage in vivo and in vitro. In addition, adenovirus-transfected primary neurons were used to investigate the possible signaling pathway involved in the neuroprotective anti-apoptotic effects of RA.

## Methods

### Animals

All animal experiments were approved by the Animal Experimentation Ethical Committee of the Zoology Center at Chongqing Medical University (Chongqing, China) and in accordance with the National Institutes of Health Guide for the Care and Use of Laboratory Animals (NIH Publication No. 8023, revised 1978). Sprague Dawley (SD) rats were procured from the Experimental Animal Center of Chongqing Medical University [SCXK (Yu) 2012–0015]. The rats were randomly assigned into four groups: control (sham), VA normal (VAN), VAD and vitamin A supplemented (VAS). Random number was generated with SPSS 17. The Animal Care Committee of Chongqing Medical University approved the experimental protocol.

### Diets

The female breeder rats in the VAD and VAN groups were fed with 300 IU and 7000 IU retinol/kg diet per day, respectively, for 4 weeks, then throughout pregnancy, and the pups were nursed from the VAD or VAN mother rats until the end of the experiment [18]. The VAS rats were VAD rats fed by VAN dams from HIBD P1 until the end of the experiment. The diets were the same except for VA content (Additional file 1: Figure S1).

### Hypoxic-ischemic animal model

The hypoxic-ischemic animal model was established using the Rice-Vannucci method [19]. The ligation of left common carotid artery was performed on 7-day-postnatal rats. One hour after the surgical procedure, the rats were put in a hypoxic tank and received hypoxic treatment (8% oxygen and 92% nitrogen) at a flow rate of 0.5 L/min for 2.5 h. The control group received the same treatment as the other groups except for the ligation and hypoxic treatment.

### HPLC testing of serum VA

The serum VA concentrations were estimated using HPLC in accordance with our previously described methods with slight modifications [20]. Two hundred microliters of serum was dissolved in 200 μL of dehydrated alcohol; 1000 μL of hexane was added and fully mixed, and the solution was centrifuged at 13,200rpm for 8 min. Then, 500 μL of the supernatant was carefully transferred and dried with nitrogen. The residue was dissolved in the mobile phase (methanol: water = 97:3). Finally, an HPLC apparatus (DGU-20As, Shimadzu Corporation, Japan) was then used to detect the prepared sample (C18, 315 nm).

### Measurement of apoptosis by TUNEL immunofluorescence staining

Rats from VAN and VAD groups were killed on post-HIBD days 3 and 7. Additionally, serial hippocampal sections were prepared, and the nuclei were stained with Hoechst 33,258 (Beyotime, China). Imaging was performed using an inverted fluorescence microscope system (NikonTE2000-S, Japan). We counted the number of TUNEL-positive cells in corresponding square regions.

### Morris water maze test

A Morris water maze test system [21] (MWM SLY-WMS 2.0, China) was used to evaluate the spatial learning and memory functions of rats, as previously described. Briefly, a visible platform was used to evaluate the rats’ vision on the first training day. Animals were exposed to an invisible platform to raise their ability of learning and memory from the second to the fifth day. In the whole 5 days, the average escape latency and path length in locating the platform were recorded. We conducted a probe trial with no escape platform and recorded the number of times that the rats swam across the former platform location in 60 s on the sixth day.

### Shuttle box test

A shuttle box test [4] (KE KE ZH-DSX2, China) was performed on post-HIBD day 30. The rats were placed in the shuttle box for 1 min to adapt to the environment and then placed in the shock zone for training on the first day. The formal test was conducted from the second to the fifth day. If the rats ran into the safe chamber within 10 s after the sound, the response was recorded as an active avoidance response; if the rats did not run into the safe chamber when given electric shock, the response was recorded as a passive avoidance response; if there was no response, the result was recorded as a no avoidance response.

### Isolation and culture of primary neurons

Zero-day-old rat pups were killed and hippocampal neurons were isolated and cultured according to previous procedures with some modifications [22]. The hippocampus of each rat was removed and digested by treatment with TrypLE (Gibco, USA) at 37°C for 30 min, and then centrifuged at 1000 rpm for 5 min to obtain the precipitate. Finally, the cells were seeded in a 6-well plate with 10% fetal bovine serum (FBS) (Gibco, USA) in DMEM/F12 medium (Gibco, USA). The medium was changed to Neurobasal medium (Gibco, USA) involving 2% B27 supplement (Gibco, USA) and 0. 5 mM L-glutamine (Gibco, USA) the next day.

### Oxygen and glucose deprivation

On the day of the experiment, the culture medium was replaced with Earle’s balanced salt solution [17] (EBSS; HyClone, USA). OGD was induced by placing the neurons in a humidified incubator (Thermo, USA) containing a mixture of 5% oxygen and 95% nitrogen for 1.5 h to simulate ischemic injury.

### RA treatment

RA (Sigma, USA) was added to the Neurobasal medium at final concentrations of 0, 1, 5, 10, 20, or 40 μmol/L for 24 h. Next, the neurons that had been treated with each concentration of RA were injured by OGD.

### Detection of apoptosis by annexin V-PI staining and flow cytometry

Each well was washed twice with D-Hank’s solution after OGD. The cells were collected and digested with TrypLE for 1 min, and centrifuged at 1000 rpm at 4°C for 5 min. The cells were measured sequentially with a flow cytometer (BD FACSAria, USA).

### Measurement of mitochondrial membrane potential by JC-1 staining and flow cytometry

Each well was washed twice with D-Hank’s solution after OGD. The cells were collected and digested with 0.5 mL TrypLE for 1 min, and centrifuged at 1000 rpm at 4°C for 5 min. The cells were measured sequentially using a flow cytometer (BD FACSAria, USA).

### Measurement of caspase-3 and caspase-8 protein activity by ELISA

Hippocampal tissue homogenates (20~40 mg) and 100 μL/100 million primary neurons were subjected to lysis on ice for 15 min. The lysates were centrifuged at 12,000 rpm at 4°C for 5 min. The caspase-3 and caspase-8 activity levels were measured at 405 nm using an automatic microplate reader ELx800 (BioTek, USA).

### RNA interference of RARα

Recombinant adenoviruses carrying the rat RARα (overRARα) or RNA interference virus RARα-siRNA (siRARα) were used to infect neurons [18]. The recombinant adenovirus was allowed to infect the neurons for 24 h to test the mRNA levels and 48 h to explore the protein levels. Red fluorescent protein (RFP) was used to label the nonspecific siRNA (siRARγ) and acted as the negative control.

### Real-time PCR RARα and other signaling pathway molecules in the hippocampus and primary neurons

Extraction of the hippocampal and primary neuronal RNA was performed using a total RNA isolation system, EZgenoTM (Genemega, USA). The purified mRNA was reverse transcribed into cDNA using the PrimeScript RT Reagent Kit (TaKaRa, Japan). cDNA quantification by real-time PCR was performed using a StepOne v2.1 Real-Time PCR instrument (ABI, USA) and RealMasterMix (SYBR Green; Tiangen Biotech, China). The cycles were performed as follows: denaturation at 95°C for 10 min, followed by 45 cycles of 95°C for 15 s, 60°C for 60 s, and 72°C for 30 s. Data were standardized to the endogenous expression of β-actin.

**Table:** 

RARα	Fwd: 5′CAGGAGGGAGAAGGCAGTGAC3′ Rev: 5′ATGGCTTGAGTTCGGAGGACAG3′
caspase-8	Fwd: 5′GGCAGCCAGTTCTTCGTT3′ Rev: 5′CTCGGCGACAGGTTACAG3′
caspase-3	Fwd: 5′GGGTGCGGTAGAGTAAGC3′ Rev: 5′CTGGACTGCGGTATTGAG3′
Bid	Fwd: 5′CCTGGAAATAGGGAGACG3′ Rev: 5′GATACGGCAAGAATTGTGAA3′
Bax	Fwd: 5′AAGTAGAAGAGGGCAACCAC3′ Rev: 5′GATGGCAACTTCAACTGGG3′
Bcl-2	Fwd: 5′CGGGAGAACAGGGTATGA3′ Rev: 5′CAGGCTGGAAGGAGAAGAT3′
PI3K	Fwd: 5′CTGGAAGCCATTGAGAAG3′ Rev: 5′CAGGATTTGGTAAGTCGG3′
Akt	Fwd: 5′CTCTTCTTCCACCTGTCTCG3′ Rev: 5′CTTGATGTGCCCGTCCTT3′
Bad	Fwd: 5′CAGGCAGCCAATAACAGT3′ Rev: 5′CCTCCATCCCTTCATCTT3′
β-actin	Fwd: 5′GCATAGCCACGCTTGTTCTTGAAG3′ Rev: 5′GAACCGCTCATTGCCGATAGTG3′

### Western blotting of RARα and other signaling pathway molecules in the hippocampus and primary neurons

The protein extracted from hippocampus or primary neuron homogenates was used for western blotting. The membranes were incubated in primary antibodies including anti-RARα (1:250, Abcam, USA), anti-β-actin (1:150, Santa Cruz, USA), anti-PI3K (1:200, Abcam, USA), anti-p-Akt (1:250, Abcam, USA), anti-Akt(1:250, Cell Signaling, USA), anti-p-Bad(1:100, Santa Cruz, USA), anti-Bad (1:200, Cell Signaling, USA), anti-caspase-3(1:100, Santa Cruz, USA), anti-caspase-8(1:100, Santa Cruz, USA), anti-Bcl-2 (1:150, Santa Cruz, USA), anti-Bax(1:100, Santa Cruz, USA), and anti-Bid(1:200, Abcam, USA) at 4 °C overnight, respectively. The blot was probed with an enzyme-linked secondary antibody (typically horseradish peroxidase) for 1h at room temperature. Then, the blot was stained with 3,3′-diaminobenzidine (DAB) (Tiangen, China) and the signals from the chemiluminescent detection reagents were photographed using an ECL Imaging System (BioRad, USA). The samples were normalized to β-actin.

### Statistical analysis

The statistical analyses were performed using one-way analysis of variance (ANOVA), repeated-measures ANOVA, the chi-squared test, and the Student-Newman-Keuls (SNK-q) test. All statistical analyses were computed in SPSS17 software by professional staff. The results are expressed as the means ± SEM. *P* ≤ 0.05 was considered significant.

## Results

### VA levels of the VAN, VAD, and VAS groups (Additional file 2: Figure S2)

The VA level assessment of VAD rats was in accordance with the standards for humans. The serum VA levels of rats were conformed to the WHO standards (1996) concerning VAN (≥1.05 μmol/L), marginal VAD (MVAD) (0.7~1.05 μmol/L), VAD (<0.7 μmol/L), and severe VAD (SVAD) (<0.35 μmol/L) status [23]. As shown in Additional file 2: Figure S2, the VAD group showed significantly decreased serum VA level relative to VAN group at every post-HIBD stage. The VAS group showed significantly higher VA level than VAD group on P7~40. The VA levels of all the groups showed increasing trends from P1 to P40, and the trends were in accordance with normal human physiological process.

### Learning ability and spatial memory of VAN, VAD, and VAS groups after HIBD (Fig. 1)

**Fig. 1:**
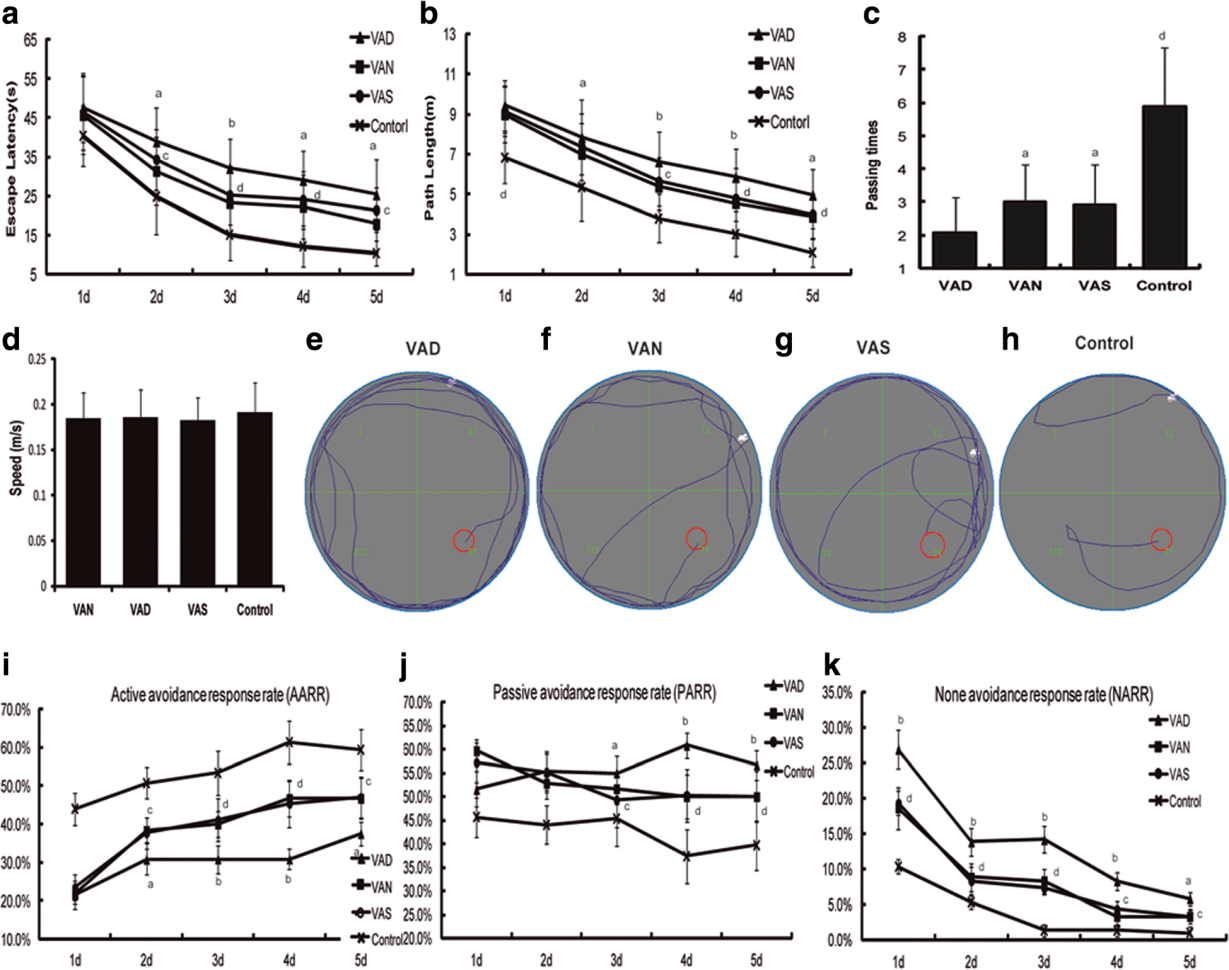
The learning ability and spatial memory of the VAD, VAN and VAS groups. **a** The escape latency of all the groups. **b** The path length of all the groups. **c** The passing times of all the groups. **d** The swimming speed levels between the four groups. **e** A representative trace of VAD group. **f** A representative trace of VAN group. **g** A representative trace of VAS group. **h** A representative trace of control group. **i** The active avoidance response rates of the four groups. **j** The passive avoidance response rates of the four groups. **k** The none avoidance response rates of the four groups. The data are expressed as the means ± SEM, *N* = 20, ^a^*P* ≤ 0.05, ^b^*P* ≤ 0.01, ^c^*P* ≤ 0.05, ^d^*P* ≤ 0.01

Figure 1 (panels **a**, **b**) shows that the escape latency and path length of all the groups gradually decreased during the test period. The VAD group showed longer escape latency and path length than VAN and VAS groups on second, third, fourth, and fifth test days. Moreover, the VAD group spent less time than VAN or VAS group in the target quadrant during the probe trial test (panel **c**). No significant difference of the swimming speed levels between the four experimental groups (panel **d**). Further, learning ability was examined through the shuttle box test. It was found that the VAD group showed a decreased active avoidance response rate (AARR) from the second to fifth test day (panel **i**), an increased passive avoidance response rate (PARR) from the third to fifth test days (panel **j**), and an increased no-avoidance response rate (NARR) on all the test days (panel **k**). Taken together, these data indicate that VAD group showed impaired learning ability and spatial memory relative to VAN group in the later post-HIBD period, and VA supplement could alleviate the impairment induced by VAD.

### Cell apoptosis in the DG, CA3, and CA1 regions of the hippocampus after HIBD (Fig. 2)

**Fig. 2:**
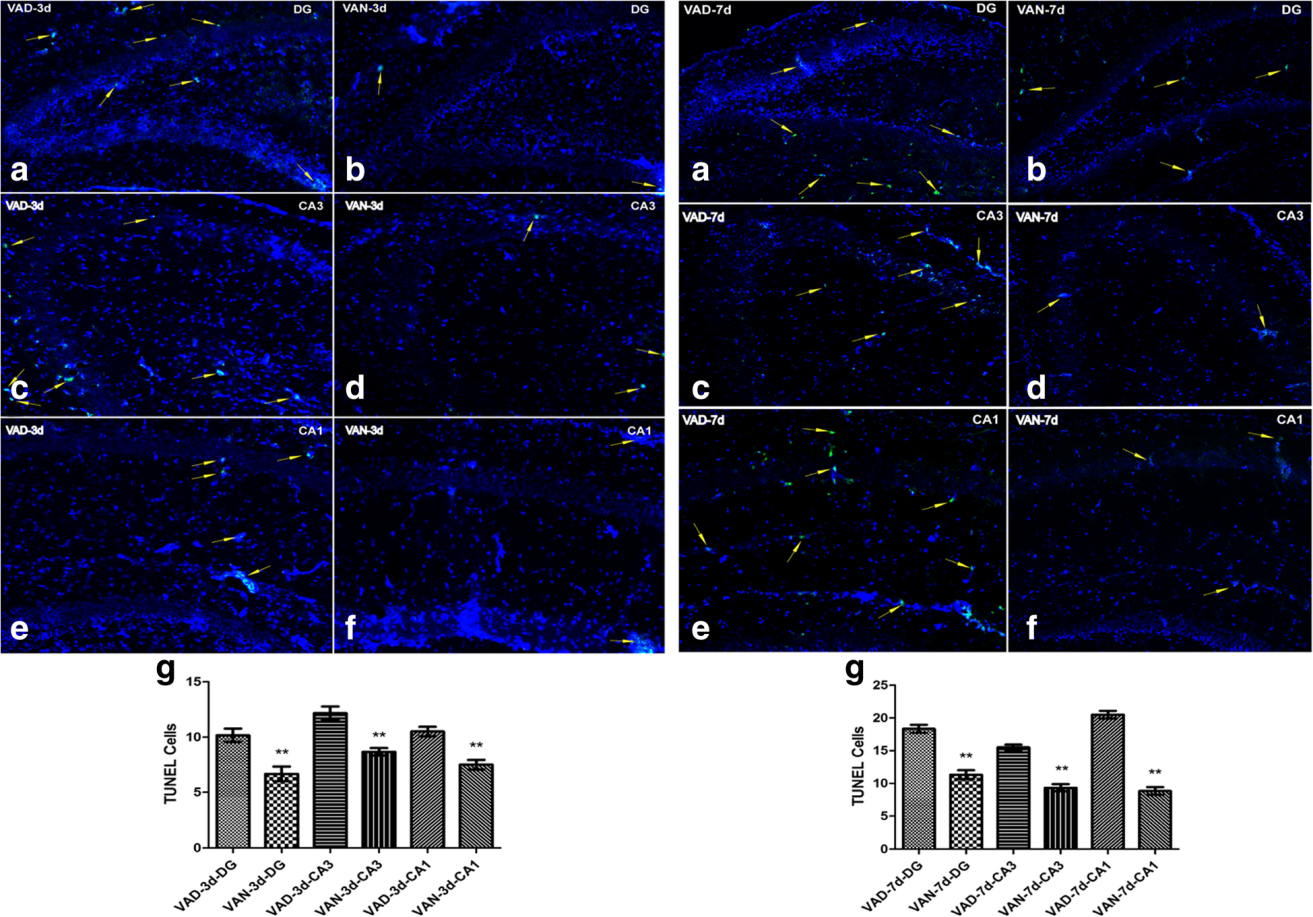
A comparison of hippocampal cell apoptosis between the VAN and VAD groups on post-HIBD days 3 and 7. **a** VAD DG area **b** VAN DG area **c** VAD CA3 area **d** VAN CA3 area **e** VAD CA1 area **f** VAN CA1 area. The yellow arrows indicate TUNEL-positive cells. **g** The number of apoptotic cells in different areas of the two groups.The data are expressed as the means ± SEM, *N* = 9, ^*^P ≤ 0.05, ^**^P ≤ 0.01

To explain why VAN group had a better neurological prognosis than VAD group after HIBD injury, apoptosis in hippocampal sections during the acute stage of HIBD was analyzed by TUNEL staining. As shown in Fig. 2, the numbers of apoptotic cells in the hippocampal DG, CA3, and CA1 regions of VAD group were significantly higher than those of VAN group (yellow arrow) on post-HIBD day 3 and day 7. The apoptotic cells were mainly located in the hippocampal pyramidal layer. The apoptosis also became more severe and began to spread from the pyramidal layer to the molecular layer. The results suggested that VAD can significantly aggravate the apoptosis of hippocampus cells in HIBD.

### RA modulates the PI3K/Akt pathway via RARα signaling to influence apoptosis in vivo (Fig. 3)

**Fig. 3:**
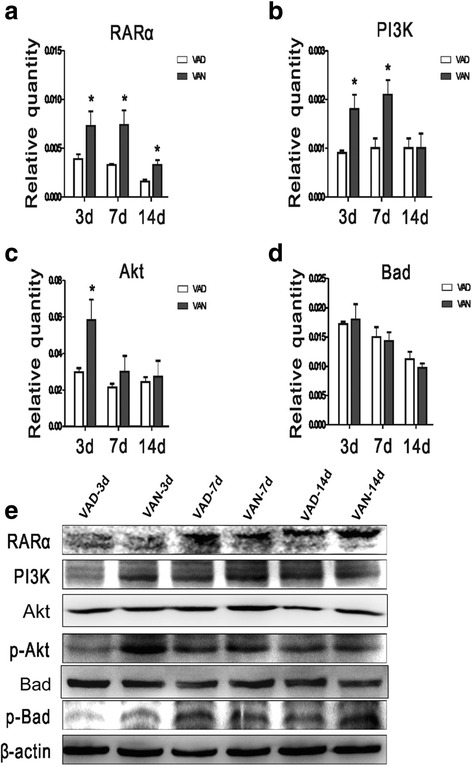
RA modulates the PI3K/Akt pathway to influence apoptosis via RARα signaling in vivo. **a** The mRNA expressions of RARα in the VAN and VAD groups on P3~ 14. **b** The mRNA expressions of PI3K in the two groups. **c** The mRNA expressions of Akt in the two groups. **d** The mRNA expression levels of Bad in the two groups. **e** The protein expression levels of RARα, PI3K, Akt, p-Akt, Bad and p-Bad between the two groups in different stages. The data are expressed as the means ± SEM, *N* = 9, ^*^P ≤ 0.05, ^**^P ≤ 0.01

Then we tested the hypothesis that the RA signaling pathway influenced apoptosis via the PI3K/Akt pathway. As shown in Fig. 3, the RARα mRNA levels of VAN group were inecreased relative to VAD group on P3~ 14 (panel **a**). The RARα protein expression level of VAN group was higher than that of VAD group on P14 (panel **e**). The PI3K mRNA and protein expression levels of VAN group were higher than those of VAD group on P3~ 7 (panels **b, e**). Similarly, the Akt mRNA expression level in VAN group was higher than that in VAD group on P3 (panel **c**), and the Akt protein expression levels in VAN group were higher than those in VAD group on P7~ 14 (panel **e**). In addition, the protein levels of phosphorylated Akt (a downstream signaling molecule of PI3K) were elevated in VAN group relative to VAD group on P3~ 7 (panel **e**). However, the mRNA levels of Bad (a molecule downstream of p-Akt) were not significantly different between two groups on P3~ 14 (panel **d**), but the phosphorylated Bad protein expression levels in VAN group were significantly higher than that in VAD group on P3~ 14 (panel **e**). These results showed that VA could activate the RARα receptor and PI3K through its active metabolite RA, and then further promote the phosphorylation of Akt and Bad.

### RA modulates Bcl-2/Bax, Bid/caspase-8, and caspase-3 to influence apoptosis in vivo (Fig. 4)

**Fig. 4:**
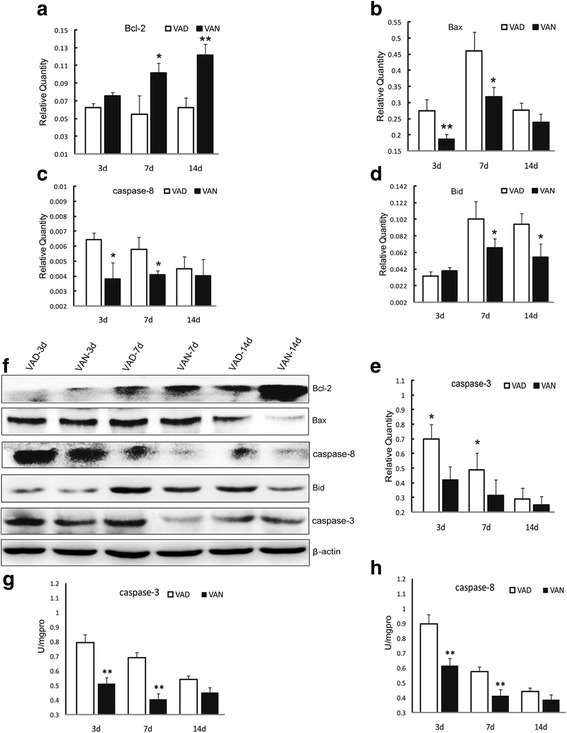
Hippocampal mRNA and protein levels of the apoptosis pathway in the VAD and VAN groups. **a** The mRNA expression levels of Bcl-2 in the VAN and VAD groups on P3~ 14. **b** The mRNA expression levels of Bax in the two groups. **c** The mRNA expression levels of caspase-8 in in the two groups. **d** The mRNA expression levels of Bid in the two groups. **e** The mRNA expression levels of caspase-3 in the two groups. **f** The protein expression levels of Bcl-2, Bax, caspase-8, Bid, and caspase-3 between the two groups in different stages. **g** The caspase-3 protein activities in the cytoplasm of the VAD and VAN groups. **h** The caspase-8 protein activities in the cytoplasm of the two groups. The data are expressed as the means ± SEM, *N* = 9, ^*^P ≤ 0.05, ^**^P ≤ 0.01

To understand the effect of RA signaling in detail, we further analyzed several mitochondrial apoptosis-associated molecules. As shown in Fig. 4, the Bcl-2 mRNA and protein expression levels in VAN group were higher than those in VAD group on P7~ 14 (panels **a, f**). The Bax mRNA levels of VAN group were lower than that of VAD group on P3~ 7 (panel **b**), and the Bax protein expression level of VAN group was lower than that of VAD group on P14 (panel **f**). In addition, the mRNA and protein expression levels of caspase-8 in VAN group were lower than those in VAD group on P3~ 7 (panels **c, f**). Similarly, the mRNA and protein expression levels of Bid (a signaling molecule downstream of caspase-8) in VAN group were lower than those in VAD group on P7~ 14 (panels **d, f**). Furthermore, it was found that the caspase-3 mRNA and protein levels of VAN group were significantly lower than those of VAD group on P3~ 7 (panels **e, f**), Finally, the differences in the protein activities of caspase-3 and caspase-8 were analyzed. The protein activities of caspase-3 and caspase-8 in VAN group were significantly lower than those in VAD group on P3~ 7 (panels **g, h**). These findings revealed that normal VA levels could affect the caspase-8/Bid and caspase-3 pathways through RA signaling and inhibit hippocampal apoptosis.

### The apoptosis rate of primary neurons injured by OGD with different concentrations of RA (Fig. 5)

**Fig. 5:**
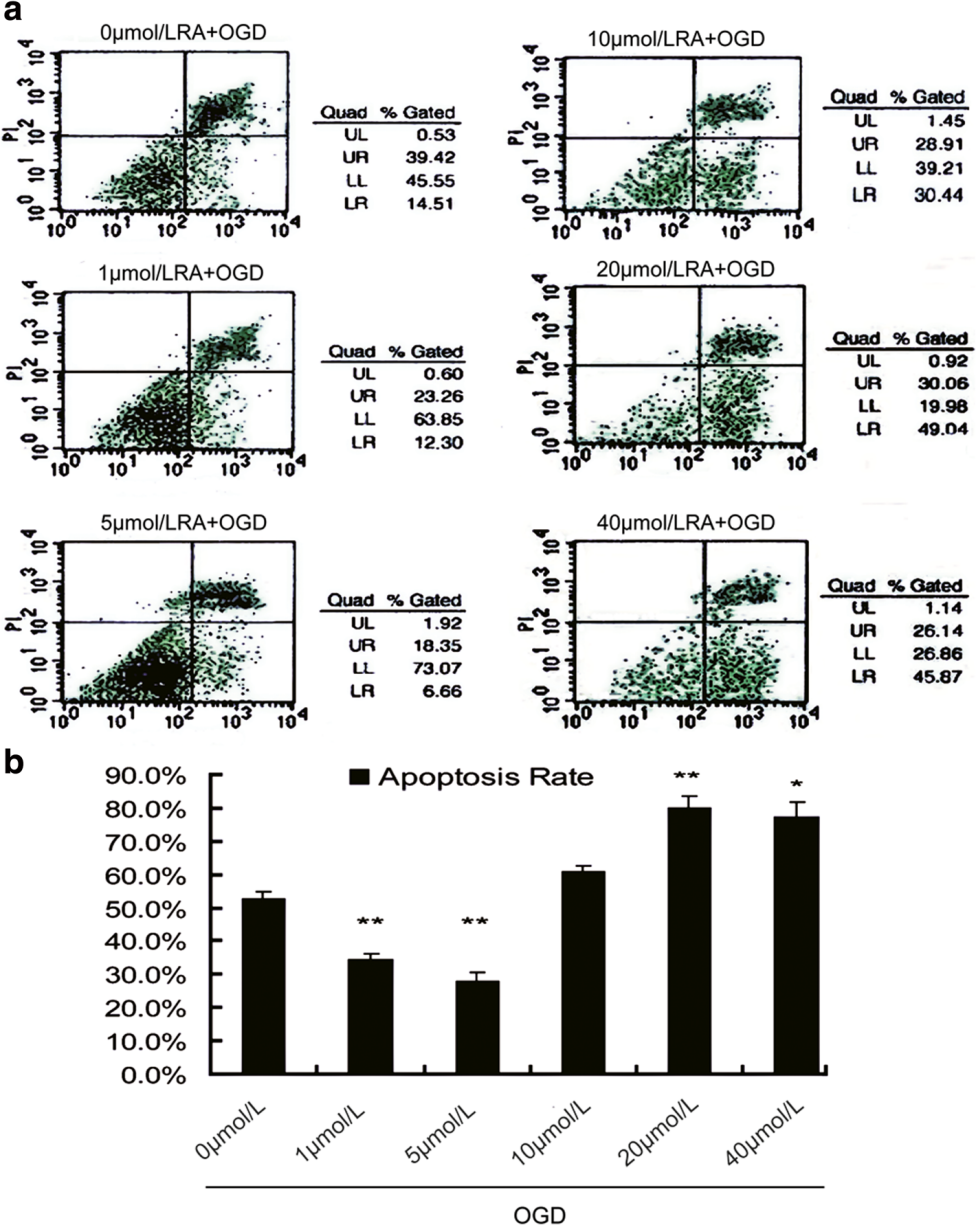
The apoptosis rate of primary neurons injured by OGD at different concentrations of RA. **a** Flow cytometry for apoptosis in primary hippocampal neurons after 0~ 40 μmol/L RA treatment. **b** A comparison of apoptosis rates for different concentrations of RA. The data are expressed as the means ± SEM, *N* = 5, ^*^P ≤ 0.05, ^**^P ≤ 0.01

The in vivo tests revealed that normal VA levels can inhibit apoptosis. Attempts were made to discover whether appropriate concentrations of RA could affect neural cell apoptosis after HIBD injury. A concentration range of 0~ 40 μmol/L of RA were used. As shown in Fig. 5, the apoptosis rates in 1~ 5 μmol/L concentration range were significantly lower than those of the other concentration groups. However, the apoptosis rates were highest at 20~ 40 μmol/L concentration range. This revealed that neuronal apoptotic protection could be modulated within a physiologically appropriate RA concentration range.

### The apoptosis rate of primary neurons injured by OGD at different RA receptor levels (Fig. 6)

**Fig. 6:**
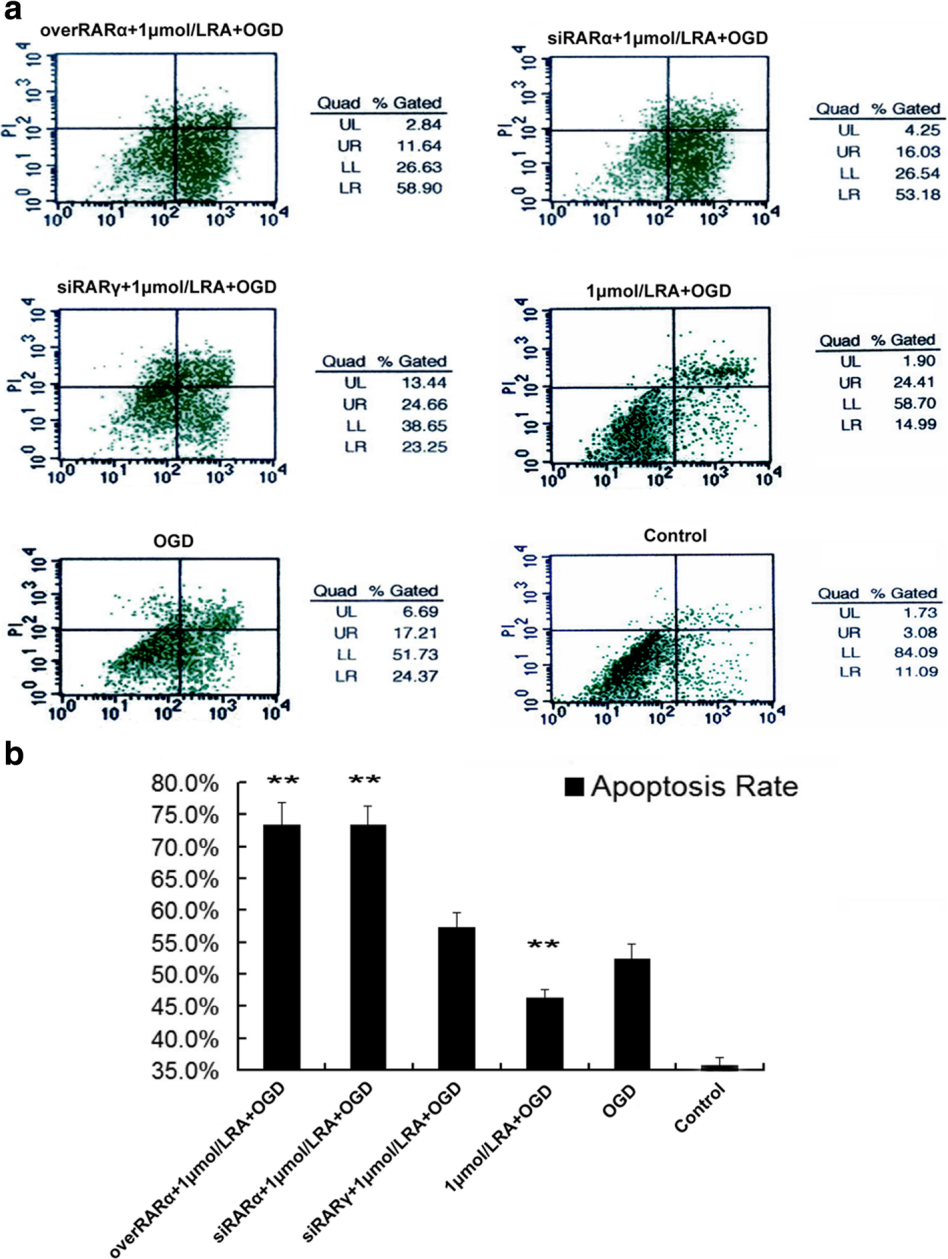
The apoptosis rate of primary neurons injured by OGD at different RA receptor levels. **a** Flow cytometry for apoptosis in primary hippocampal neurons at different RA receptor levels. The UR (upper right) and LR (lower right) quadrants represent apoptotic cells. **b** A comparison of apoptosis rates for the different RA receptor levels. The data are expressed as the means ± SEM, *N* = 5, ^*^P ≤ 0.05, ^**^P ≤ 0.01

The aforementioned findings showed that RA within a suitable concentration range (1~ 5 μmol/L) protected the neurons from apoptosis. As shown in Fig. 6, the apoptosis rate of 1 μmol/L RA + OGD group was lower than that of OGD group. And the apoptosis rates in overRARα + 1 μmol/L RA + OGD and siRARα + 1 μmol/L RA + OGD were significantly higer than those of siRARγ + 1 μmol/L RA + OGD group (negative transfection group), which suggest that overexpression and silencing of RARα significantly promoted apoptosis in primary neurons after OGD injury. These results revealed that a moderate level of the RA signal is required to produce an anti-apoptotic effect at the ligand and receptor levels.

### The rate of abnormal mitochondrial membrane potential (MMP) in primary neurons injured by OGD at different RA receptor levels (Fig. 7)

**Fig. 7:**
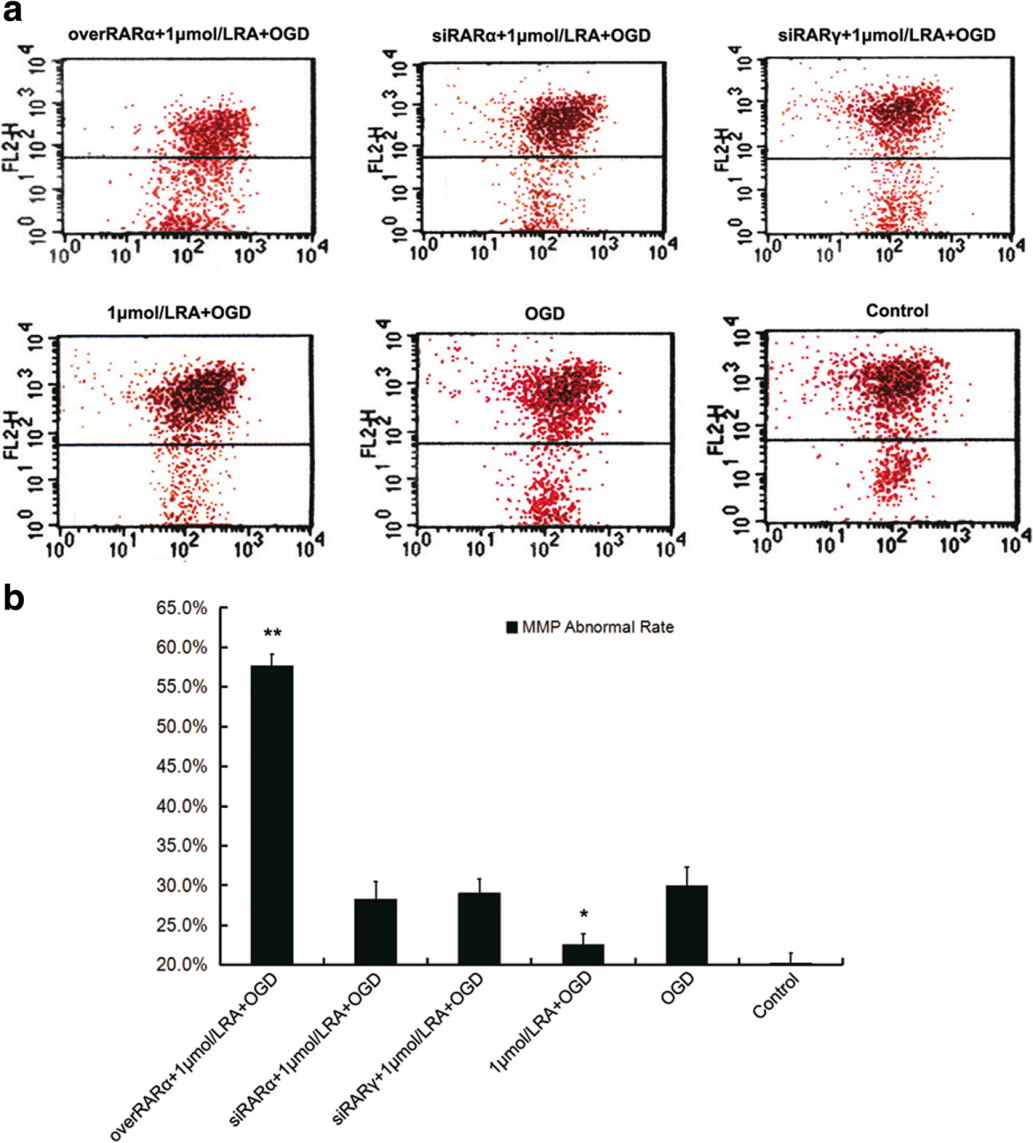
The rate of abnormal mitochondrial membrane potential in primary neurons injured by OGD at different RA receptor levels. **a** Flow cytometric measurement of the rate of abnormal MMP at different RA receptor levels. The lower quadrants represent cells with abnormal MMP. **b** A comparison of the rates of abnormal MMP for the different RA receptor levels. The data are expressed as the means ± SEM, *N* = 5, ^*^P ≤ 0.05, ^**^P ≤ 0.01

In vivo tests have shown that RA signals can affect multiple signaling pathways, and these signaling pathways are related to mitochondrial apoptosis pathways, such as through MMP. As shown in Fig. 7, the rate of abnormal MMP in overRARα + 1 μmol/L RA + OGD group was the highest of all groups. And abnormal MMP rate in 1 μmol/L RA + OGD group was significantly lower than that in OGD group or other damage groups. This result revealed that a suitable level of RA signal mitigated MMP abnormalities.

### RA modulates the PI3K/Akt pathway to influence apoptosis via RARα signaling in vitro(Fig. 8)

**Fig. 8:**
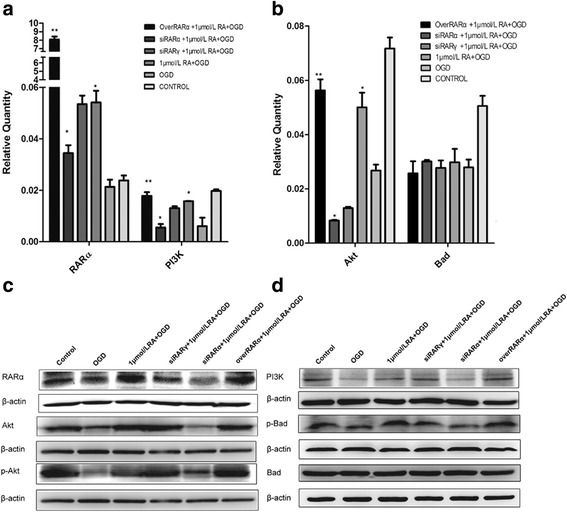
mRNA and protein levels of the PI3K/Akt signaling pathway in primary hippocampal neurons after OGD injury. **a** With different RA receptor levels, the mRNA expressions of RARα and PI3K in the different groups. **b** With different RA receptor levels, the mRNA expressions of Akt and Bad in the different groups. **c**, **d** With different RA receptor levels, the protein expression levels of RARα, PI3K, Akt, p-Akt, p-Bad and Bad among all the intervention groups. The data are expressed as the means ± SEM, *N* = 5, ^*^P ≤ 0.05, ^**^P ≤ 0.01

As shown in Fig. 8, different RARα expression levels were used to simulate different levels of RA signaling. The mRNA and protein expression levels of RARα and PI3K in overRARα + 1 μmol/L RA + OGD group and siRARα + 1 μmol/L RA + OGD group were, respectively, significantly higer and lower than those in siRARγ + 1 μmol/L RA + OGD group. The mRNA and protein expression levels of RARα and PI3K in 1 μmol/L RA + OGD group were higher than those in OGD group (panels **a, c**). The mRNA and protein expression levels of Akt and p-Akt, which are downstream of PI3K, were also similar (panels **b, c**). In addition, no significant difference was noted in mRNA expression of Bad (the p-Akt downstream molecule) among the injury groups, but the protein level of p-Bad, which is activated by p-Akt, was similar to that of PI3K and p-Akt (panels **b, d**). This demonstrated that activation of PI3K via RA signaling can promote the phosphorylation of Akt and further upregulate phosphorylation of Bad. Finally, Bad was retained in the cytoplasm and inhibited the activation of the mitochondrial apoptotic pathway. Overexpression of RARα yielded the highest PI3K/Akt activation, but this contrasted with the change found in the apoptosis rate of primary neurons.

### RA modulates Bcl-2/Bax, Bid/caspase-8, and caspase-3 to influence apoptosis in vitro (Fig. 9)

**Fig. 9:**
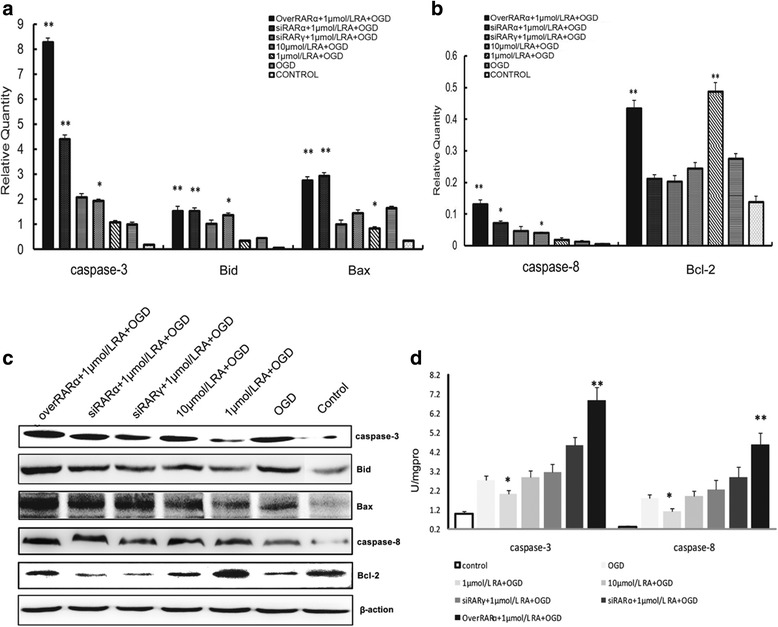
mRNA and protein levels of apoptosis-related signaling molecules in primary hippocampal neurons after OGD injury. **a** With different RA receptor levels, the mRNA expressions of caspase-3, Bid and Bax in the different groups. **b** The mRNA expression levels of caspase-8 and Bcl-2 in different groups. **c** The protein expression levels of caspase-3, Bid, Bax and caspase-8 between the different groups. **d** The protein activities of caspase-3 and caspase-8 between the different groups. The data are expressed as the means ± SEM, *N* = 5, ^*^P ≤ 0.05, ^**^P ≤ 0.01

Interestingly, Fig. 8 shows that a very high RA signal significantly upregulated the PI3K/Akt pathway but did not have a strong anti-apoptotic effect, suggesting that a very high RA signal may significantly activate the other apoptotic signaling pathways. As shown in Fig. 9, The mRNA and protein expressions of caspase-3 in overRARα + 1 μmol/L RA + OGD group and siRARα + 1 μmol/L RA + OGD group were higher than those in siRARγ + 1 μmol/L RA + OGD group. Caspase-8, Bid, Bax were also similar. No differences between the 1 μmol/L RA + OGD and OGD groups were found for the mRNA caspase-3, caspase-8 and Bid. However, the protein expressions of caspase-3, Bid, Bax were lower in 1 μmol/L RA + OGD than those in OGD group. The 1 μmol/L RA + OGD group had higher Bcl-2 mRNA and protein expressions than those of OGD group. And the protein activities of caspase-3 and caspase-8 in 1 μmol/L RA + OGD group were significantly weaker than those in OGD group. This suggests that RA could directly affect Bcl-2/Bax expression but did not directly affect caspase-3, caspase-8 or Bid expression. Instead, it impacted the activities of caspase-3 and caspase-8, which affected cleavage of Bid and its translocation from the cytoplasm to the mitochondrial membrane. This explains why low and high RA signals can significantly promote apoptosis and why the effect of overexpression was the strongest. Although the overexpression group had the highest activation of anti-apoptotic PI3K/Akt signaling, we noted that Bax, Bid/caspase-8, and caspase-3 expression and activation were also highly promoted. Therefore, the result was a strong pro-apoptotic effect.

## Discussion

### VA affects long-term neurological function and hippocampal apoptosis after the acute stage of HIBD

HIBD is a brain lesion in perinatal newborns that is caused by hypoxia and decreased cerebral blood perfusion due to neonatal intrauterine asphyxia and anoxia. The global incidence rate is approximately 1/1000 to 3/1000 in full-term infants, with 15–20% mortality. Approximately 25–30% of the survivors have permanent neural defects such as cerebral palsy, epilepsy, memory deficiency, and hypophrenia. No treatment is currently available, and the pathogenesis of this condition is still not clear [24–26]. The main outcome of HIBD involves neuronal apoptotic processes that spread beyond the immediate ischemic regions [27, 28].

Sufficient VA is required for normal embryonic development and postnatal tissue homeostasis, including cell proliferation, tissue differentiation, immunoregulation, and organogenesis. VAD can modify the structure of the macromolecular components of extracellular matrix, and such alterations potentially leads to organ dysfunction and diseases [29]. Moreover, VAD in gestation and early life leads to spatial learning and memory lesions in adolescence: this process involves the molecular interactions of retinoid acid nuclear receptor α (RARα) [18]. RA acts as a ligand to integrate with RA receptors and receptor-specific nuclear receptor response elements, switching transcription factors from potential repressors to transcriptional stimulators. RA plays a pivotal role in early phases of neurogenesis, neuronal survival, and synaptic plasticity, and is an essential contributor during development that enables proper cognitive function in adolescence [30–32].

A previous study elucidated that VAD could inhibit learning and spatial memory retrieval via the RARα signaling pathway in the early stage following HIBD [4]. Our results showed that the VAN and VAS groups had advantages in learning and spatial memory after HIBD (Fig. 1). The findings indicate that suitable VA nutritional status is beneficial for the neurological function of newborns with HIBD, and perhaps VA should be included as part of the standard HIBD clinical intervention. Zhu found that RA reduced infarct size and cardiomyocyte apoptosis in myocardial ischemia/reperfusion (I/R) injury [33]. Kong suggested that RA may serve as a new therapeutic approach to prevent blood brain barrier (BBB) dysfunction and tPA-induced rat ICH in ischemic stroke, and the protective effect of RA on the BBB was dependent on RARα [34]. The hippocampal TUNEL test demonstrated that VAN decreased the apoptosis in the CA1, CA3, and DG regions at the acute stage of HIBD. This finding confirmed the histological results (Fig. 2).

### RA signal mediates mitochondrial apoptosis depending on signaling intensity

The in vivo experiment showed that rats with normal physiological levels of VA had better learning and spatial memory than rats with VAD, and the advantage was associated with lower levels of apoptosis in the hippocampus. A number of studies have suggested that an excess of VA can affect neurological development. RA can promote carcinoma cell apoptosis, and large doses of RA are currently applied therapeutically for certain types of cancer [12]. However, RA inhibits the apoptosis of neural cells in rat ischemic stroke [34]. Wang revealed that all-trans retinoic acid (1~ 10 μmol/L) inhibited cobalt chloride-induced apoptosis in PC12 cells [35]. Upon in depth analysis, it was found that higher doses of RA caused the apoptosis of immune cells and tumors, whereas a smaller dose was required for protection from ischemia in the nerves and myocardial tissue. It is speculated that appropriate RA signaling could inhibit the apoptosis of hippocampal neurons in HIBD. In vitro tests supported the hypothesis that 1~ 5 μmol/L was the optimal concentration range for anti-apoptotic effects; excessively high or low dose were pro-apoptotic (Fig. 5). Moreover, it was demonstrated through overexpression and silencing of RARα that suitable RA signaling is necessary for the anti-apoptotic effect (Fig. 6). Furthermore, it was found that RA can affect the initial events of mitochondrial apoptosis (MMP). Appropriate RA signaling showed the lowest rate of an abnormal MMP, which was consistent with previous findings [16, 17].

### Suitable RA signaling upregulated the PI3K/Akt/Bad and Bcl-2/Bax pathways and downregulated the caspase-8/Bid pathway to protect MMP and thereby inhibit mitochondrial apoptosis

The molecular mechanisms that underlie the effect of RA on hippocampal neuronal apoptosis in HIBD are not clear. Currently, activation of the PI3K/Akt signaling pathway is known to be related to neuronal survival and anti-apoptotic effects after ischemic damage. Zhang et al. and Huang et al. found that increased production and activation of PI3K/Akt can antagonize the apoptosis induced by OGD injury [36, 37]. The neuroprotective mechanisms of Ginsenoside Rb1 and the defense of mesenchymal stromal cells against neuronal apoptosis produced by an ischemic insult occurred through the activation of PI3K/Akt signaling [38, 39]. Accumulating evidence has revealed that PI3K/Akt plays a principal role in the resistance against neural injury. However, a number of studies have shown that RA regulates the PI3K/Akt signaling pathway in some types of cells. Uruno et al. demonstrated that RA played an important role in vascular endothelial cells through RAR-mediated PI3K/Akt pathway activation, in which nitricoxide was produced to resist vascular disease in the event of an endothelial insult [40]. This has been demonstrated in vivo (Fig. 3) and in vitro (Fig. 8).

Bad (Bcl-associated death protein) is a recognized pro-apoptotic protein, and the active form is phosphorylated on its serine residues. Free Bad may cause a decrease in MMP via translocation from the cytosol to the mitochondrial membrane to displace pro-apoptotic Bax (Bcl-2-associated X protein) from the anti-apoptotic Bcl-XL, and Bax subsequently translocates to the mitochondrion and induces cytochrome c release and caspase activation. Bad loses this pro-apoptotic effect after it is phosphorylated and combines with the cytosolic protein 14–3-3 [1, 2]. The phosphorylation of Bad is the target of the PI3K/Akt pathway. Neuregulin-1 upregulates p-Bad through activation of the PI3K/Akt signaling pathway to restrain neuronal apoptosis after transient focal cerebral ischemia [41]. Additionally, astaxanthin upregulates the phosphorylation of Akt/Bad, thus activating the Akt/Bad signaling pathway to dramatically decrease neuronal apoptosis in the early stages of brain damage [42]. In the present study, the expression of PI3K/p-Akt/p-Bad was significantly enhanced in RA-treated primary neurons (Fig. 8) and VAN rats (Fig. 3). Interestingly, the effects on the trends in the PI3K, p-Akt, and p-Bad expression levels were consistent with the changes in the expression level of RARα after infection of OGD-injured neurons with adenovirus (Fig. 8). However, this process depends on the phosphorylation of Bad protein and cannot impact the transcription of the Bad gene. The preliminary results demonstrated that RA regulated the downstream PI3K/Akt signaling cascade via RARα signaling to accelerate the survival of cultured primary neuronal cells and prevent apoptosis, and the effect was dependent on the signal intensity.

Interactions between the Bcl-2 family proteins determine whether cells live or die. The expression levels of Bcl-2 and Bax and the ratio of Bcl-2 to Bax are important factors in mitochondrial apoptosis [43]. Meanwhile, the expression of Bcl-2 and CCND1 was enhanced. In contrast, URG4/URGCP and Bax gene expression declined significantly [44]. The in vivo results demonstrated that the VAN rats had higher Bcl-2 and lower Bax expression than the VAD rats (Fig. 4). The in vitro data showed that suitable RA signal intensity promoted Bcl-2 and inhibits Bax expression, thereby increasing the ratio of Bcl-2 to Bax (Fig. 9). Thus, it suppresses the decrease of MMP (Fig. 7).

The caspase-8/Bid pathway is also an important signaling cascade that impacts MMP in mitochondrial apoptosis [45]. Caspase-3 is the pivotal signal of the apoptotic pathway in neuronal cells [46]. The in vivo results of the present study revealed that the VAN group had lower caspase-8/Bid and caspase-3 expression or activity than the VAD group (Fig. 4). In addition, the in vitro data showed that a suitable RA signal intensity restrained the expression and activity of caspase-8 and caspase-3 (Fig. 9). Interestingly, only a certain optimal range of RA signals significantly inhibited the expression or activity of caspase-8/Bid and caspase-3. Although the overexpression of RARα activates the PI3K/Akt/Bad pathway and Bcl-2, upregulation of caspase-8/Bid and caspase-3 leads to pro-apoptotic signals. Therefore, the balance of these signals is very important in the control of hippocampal apoptosis after HIBD.

## Conclusions

In conclusion, after HIBD, sustained VAD caused underexpression of RARα, which downregulated PI3K/Akt/Bad and Bcl-2 signaling. The Bax, caspase-8/Bid, and caspase-3 pathways were also upregulated to reduce MMP and activate mitochondrial apoptosis, ultimately producing deficits in active learning and spatial memory in adolescence. VAS can partly repair the deficit. Meanwhile, excessively high or low RA signals can promote mitochondrial apoptosis. RA signaling bio-modulates mitochondrial apoptosis depending on the signal intensity. A high RA signal activated the PI3K/Akt/Bad pathway which failed to produce anti-apoptotic signals because caspase-8/Bid and caspase-3 signaling was upregulated. These findings suggest that clinical interventions for newborns with HIBD should include a suitable dosage of VA.

## Additional files

Additional file 1 **Figure S1.** (A) HIBD newborns (50 cases): serum VA is 0.474 μmol/L; newborns with neonatal pneumonia (65 cases): 0.761 μmol/L; normal newborns (15 cases): 0.844 μmol/L (^**^P ≤ 0.01, ^*^P ≤ 0.05, one-way ANOVA). (B) HIBD children with VAD: The incidence of VAD (87.8%) was significantly higher than that in children with pneumonia (40%) (^**^P ≤ 0.01, ^*^P ≤ 0.05, chi-squared test). (C) Newborns over 7 days old had no significant difference in VA level compared with newborns under 7 days old, but the VA level was significantly higher in neonatal pneumonia cases and in normal newborns (^**^P ≤ 0.01, ^*^P ≤ 0.05, one-way ANOVA). (TIFF 1976 kb)

Additional file 2 **Figure S2.** (A) The experimental flow chart. (B) The special feed formulations for the vitamin A deficiency (VAD) and normal (VAN) groups. (C) The vitamin A level of the VAD, VAN, VA supplement (VAS), and control groups during the course of the study. The VA level of VAN rats (*N* = 50) was significantly higher than that of VAD rats (*N* = 50) at every stage after HIBD (^**^P ≤ 0.01, ^*^P ≤ 0.05, SNK). The VA level of VAS rats (*N* = 50) was significantly higher than that of VAD rats (*N* = 50) on post-HIBD days 7–40(P7–P40) (^**^P ≤ 0.01, ^*^P ≤ 0.05, SNK). The VA levels of all the groups had an increasing trend from P1 to P40. The data are expressed as the means ± SEM. (TIFF 3295 kb)

## Abbreviations

HIBD: Hypoxic-ischemic brain damage
MMP: Mitochondrial membrane potential
RARE: Retinoic acid response element
OGD: Oxygen-glucose deprivation
RA: Retinoic acid
RARα: Retinoid acid nuclear receptor α
VA: Vitamin A
VAD: Vitamin A deficiency
VAN: Vitamin A normal
VAS: Vitamin A supplemented
