# Supplementary figures and images for: Vitamin A bio-modulates apoptosis via the mitochondrial pathway after hypoxic-ischemic brain damage
Source: Mol Brain. 2018 Mar 13;11:14. doi: 10.1186/s13041-018-0360-0 (PMC5851324; doi:10.1186/s13041-018-0360-0)

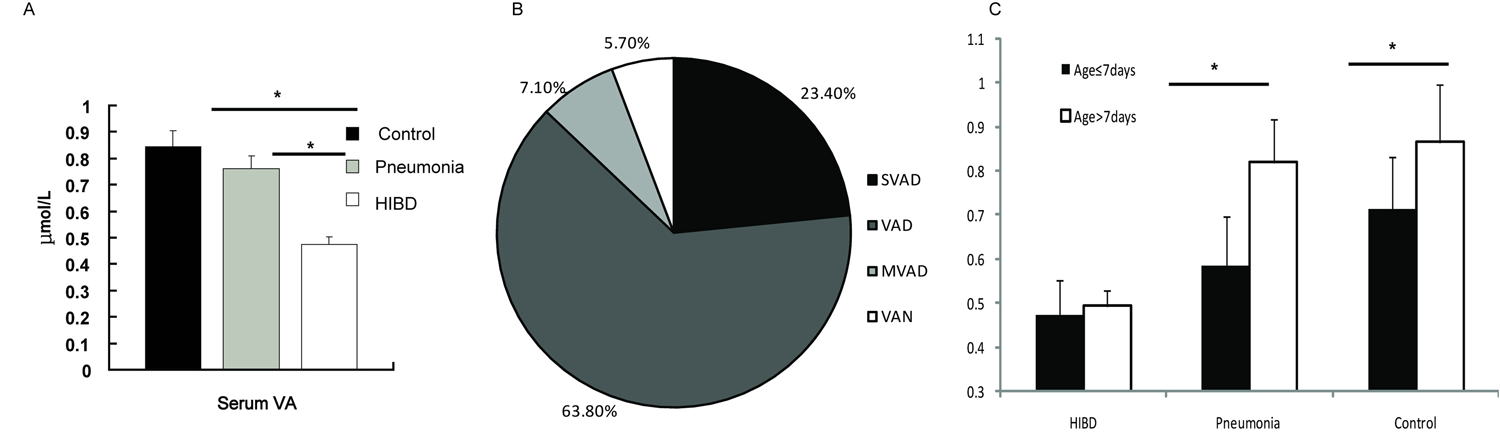

Supplement: Supplementary file 1 — Figure S1. (A) HIBD newborns (50 cases): serum VA is 0.474 μmol/L; newborns with neonatal pneumonia (65 cases): 0.761 μmol/L; normal newborns (15 cases): 0.844 μmol/L (**P ≤ 0.01, *P ≤ 0.05, one-way ANOVA). (B) HIBD children with VAD: The incidence of VAD (87.8%) was significantly higher than that in children with pneumonia (40%) (**P ≤ 0.01, *P ≤ 0.05, chi-squared test). (C) Newborns over 7 days old had no significant difference in VA level compared with newborns under 7 days old, but the VA level was significantly higher in neonatal pneumonia cases and in normal newborns (**P ≤ 0.01, *P ≤ 0.05, one-way ANOVA). (TIFF 1976 kb) [file 13041_2018_360_MOESM1_ESM.tif]

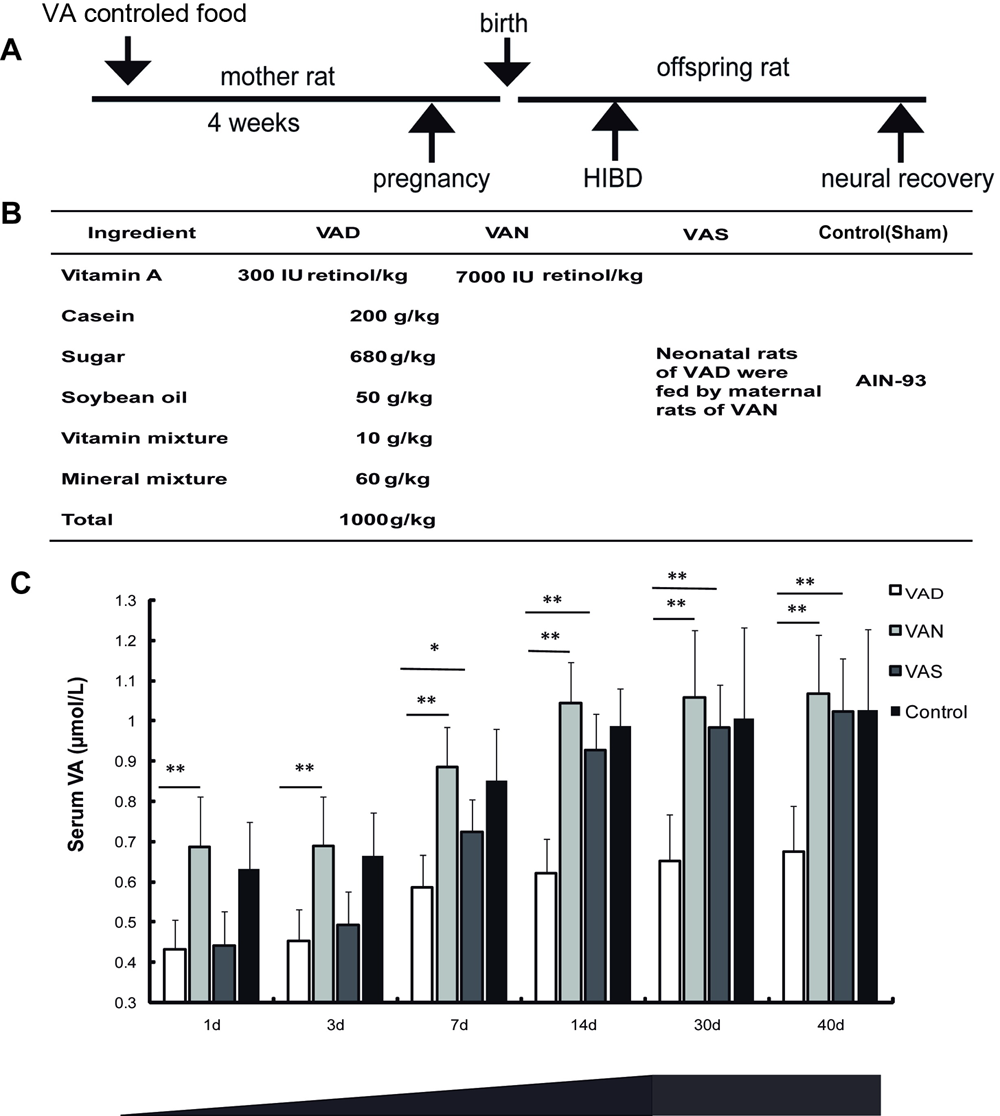

Supplement: Supplementary file 2 — Figure S2. (A) The experimental flow chart. (B) The special feed formulations for the vitamin A deficiency (VAD) and normal (VAN) groups. (C) The vitamin A level of the VAD, VAN, VA supplement (VAS), and control groups during the course of the study. The VA level of VAN rats (N = 50) was significantly higher than that of VAD rats (N = 50) at every stage after HIBD (**P ≤ 0.01, *P ≤ 0.05, SNK). The VA level of VAS rats (N = 50) was significantly higher than that of VAD rats (N = 50) on post-HIBD days 7–40(P7–P40) (**P ≤ 0.01, *P ≤ 0.05, SNK). The VA levels of all the groups had an increasing trend from P1 to P40. The data are expressed as the means ± SEM. (TIFF 3295 kb) [file 13041_2018_360_MOESM2_ESM.tif]
